# The impact of caplacizumab in the treatment of immune thrombotic thrombocytopenic purpura. A retrospective monocentric cohort study

**DOI:** 10.3389/fphar.2026.1761391

**Published:** 2026-05-15

**Authors:** Uros Markovic, Gabriele Sapuppo, Sara Frazzetto, Stephanie Grasso, Andrea Duminuco, Giuliana Giunta, Marianna Calagna, Benedetta Esposito, Manlio Fazio, Antonella Nardo, Lara Gullo, Gabriella Santuccio, Chiara Maria Catena Sorbello, Dario Leotta, Salvatore La Penta, Francesco Di Raimondo, Gaetano Giuffrida

**Affiliations:** 1 Division of Haematology and Bone Marrow Transplantation, Azienda Ospedaliero-Universitaria Policlinico G. Rodolico – San Marco, Catania, Italy; 2 Regional Reference Center for the Prevention, Diagnosis, and Treatment of Rare Congenital Disease in Children and Adult Patients, Catania, Italy; 3 Rare Hematological Disease Unit, Azienda Ospedaliero-Universitaria Policlinico G. Rodolico – San Marco, Catania, Italy; 4 Postgraduate School of Hematology, University of Catania, Catania, Italy

**Keywords:** caplacizumab, immune thrombotic thrombocytopenic purpura, platelet, thrombotic microagiopathy, treatment

## Abstract

**Background:**

Immune-mediated thrombotic thrombocytopenic purpura (iTTP) is a rare, life-threatening disorder caused by severe ADAMTS13 deficiency, leading to microvascular platelet-rich thrombi, hemolysis, and organ damage, mainly involving brain, heart, kidneys, and intestines. Standard treatment includes plasma exchange (PEX) and immunosuppressants. Caplacizumab (CAPLA), an anti-von Willebrand factor nanobody, was recently introduced into clinical practice.

**Methods:**

We retrospectively analyzed 81 patients diagnosed with iTTP at our center from 1990 to 2024, accounting for 165 acute episodes requiring treatment. We compared outcomes between episodes treated with standard of care (SOC) and those with CAPLA, evaluating number of PEX sessions to platelet normalization, hospitalization length, CAPLA use, and disease stage (new onset vs. relapse). CAPLA was administered from episode onset until 30 days after PEX suspension.

**Results:**

Median age at first episode was 39.9 years (range 13–75), with 63% female patients. Over a median follow-up of 6.4 years, 47 patients (58%) experienced at least one relapse (range 1–7), all treated with PEX and corticosteroids. Of the 165 episodes, 39 (25.5%) were treated with CAPLA, 126 (74.5%) with SOC. Baseline characteristics were similar. Median PEX sessions were significantly lower in the CAPLA group (6 vs. 9.5; p < 0.0001), as were hospitalization days (8 vs. 13.5; p < 0.0001). Exacerbation after PEX occurred in 11 patients, more frequently in the CAPLA group (13% vs. 5%), associated with higher inhibitor levels (66 U/mL). Interestingly, patients who relapsed later had lower inhibitor levels at baseline (34 vs. 63 U/mL; p = 0.045) but longer hospital stays (12 vs. 10 days; p = 0.03), possibly due to less frequent CAPLA use (18% vs. 30%).

**Conclusion:**

Caplacizumab significantly reduces PEX sessions and hospitalization duration, leading to faster clinical response, regardless of disease stage or inhibitor level. However, its use may be linked to a higher rate of exacerbation, potentially due to earlier PEX discontinuation.

## Introduction

Immune-mediated or acquired thrombotic thrombocytopenic purpura (iTTP) is a rare, life-threatening disease caused by a severe ADAMTS13 deficiency (<10%), a metalloproteinase of von Willebrand factor (vWF). The pathogenetic mechanism is based on the production of autoantibodies directed against ADAMTS13. The lack of ADAMTS13 leads to the aggregation of platelets by ultra-large VWF multimers and results in the formation of platelet-rich thrombi, with subsequent microvascular obstruction, thrombocytopenia, mechanical hemolysis, and end-organ ischemia. Organ ischemia most commonly affects brain, heart, kidney, and gastrointestinal tract. iTTP is a medical emergency associated with high morbidity and mortality (Joly et al., 2017; Sukumar et al., 2021). A prompt diagnosis and an appropriated treatment are fundamental to reduce the death rate from 90% to 5%–20%. In fact, death and severe complications usually occur during the initial days after the onset of the disease (del Río-Garma et al., 2022).

For decades, the standard of care for iTTP was based on the association of daily therapeutic plasma exchange (PEX) and immunosuppressive therapy with corticosteroids (Page et al., 2017). The humanized anti-CD20 monoclonal antibody rituximab has also been used for iTTP treatment, particularly in patients with a suboptimal response to conventional therapy, defined as refractoriness or recurrence of thrombocytopenia during the period of daily PEX or within 30 days from PEX stop. The aim of rituximab treatment in iTTP is to suppress the production of B-lymphocyte-based anti-ADAMTS13 autoantibodies (Scully et al., 2007; Scully et al., 2011; Froissart et al., 2012; Page et al., 2016).

The recent introduction in clinical practice of the nanobody caplacizumab has changed this therapeutic approach. In fact, the International Society on Thrombosis and Haemostasis now recommends the early use of caplacizumab to treat acute iTTP, in combination with PEX and corticosteroids (Zheng et al., 2020).

Caplacizumab is a humanized nano-antibody that acts by targeting the A1 domain of vWF and therefore blocking the interaction between vWF and platelets (Duggan, 2018). Caplacizumab was approved by the European Medicines Agency for iTTP in 2018, based on the results of two randomized clinical trials comparing the standard of care with and without caplacizumab (Peyvandi et al., 2016), (Scully et al., 2019). In both trials, the administration of caplacizumab was associated with faster clinical response in terms of platelet count recovery and fewer exacerbations.

After the drug’s approval, data from real-world clinical settings have been published, confirming the safety and efficacy of caplacizumab. Some concerns about the use of this drug remain in terms of cost and cost-effectiveness (Goshua et al., 2021). In this study, we retrospectively analyzed data of iTTP patients treated at our institution in the last 30 years, to understand better the clinical impact of caplacizumab use in terms of efficacy and safety in a real-world setting. Furthermore, we evaluated the role of rituximab as an immunosuppressive drug in iTTP patients.

## Matherials and methods

### Patients selection

Medical records of 81 patients diagnosed with iTTP and followed between January 1990 and June 2024 at the Hematology and Bone Marrow transplantation Unit of Azienda Ospedaliera Policlinico G. Rodolico San Marco of Catania were screened for the purpose of retrospective study enrollment. All patients with a confirmed diagnosis of iTTP who were admitted to our Center and initiated specific treatment following the diagnosis were included in this analysis.

In accordance with international guidelines, the diagnosis of iTTP was confirmed by measurement of ADAMTS13 activity below 10% and the presence of anti-ADAMTS13 IgG assay. The measurement of ADAMTS13 activity has been available in the routine clinical practice of the center since 2016. Before these methods became available in clinical practice, the diagnosis of iTTP was based on laboratory parameters, including anemia, positive hemolysis markers, a negative Coombs test, thrombocytopenia, and the presence of schistocytes on the peripheral blood smear.

We therefore reviewed all medical records of patients diagnosed with iTTP and included in the analysis only those cases in which the diagnosis of iTTP was considered reasonably certain.

The following factors were evaluated at the time of diagnosis and at each disease recurrence: age, sex, sign and symptoms of TTP, Hb levels, platelet count, comorbidities, ADAMTS13 activity, anti-ADAMTS13 IgG level, Plasmic score, number of PEX sessions required to obtain platelet normalization, number of days of hospitalization in the in-patient setting, type and duration of immunosuppressive treatment. The PLASMIC score was calculated at the time of diagnosis in patients who presented with their first episode after 2017, the year of the score’s publication. For patients diagnosed prior to that date, the score was retrospectively calculated based on data from disease onset. Forty-five patients experienced a relapse of iTTP after the initial episode and, considering both diagnosis and disease relapses, data from 165 acute iTTP clinical episodes requiring standard treatment were collected. Therefore, two different cohorts were analyzed and compared: one cohort of patients that were treated without caplacizumab, i.e., the standard of care (SOC) cohort with 126 cases, and a cohort of patients that received caplacizumab as part of iTTP treatment, the CAPLA cohort with 39 cases. Eleven patients who experienced relapses, accounting for a total of 43 iTTP episodes, were included in both cohorts as they were initially treated without caplacizumab and subsequently with caplacizumab, depending on drug availability and clinical indication for its use. Furthermore, acute episodes were divided based on disease stage between newly-diagnosed and relapsed ones in order to evaluate potential differences, including the effect of caplacizumab use. Mann-Whitney test was used for evaluation of statistical significance of continuous variables. The decision to evaluate different episodes occurring in the same patient as distinct events was made by the study authors in order to better assess the role of caplacizumab in iTTP, despite the fact that, from a methodological standpoint, this represents a limitation of the statistical analysis. However, the rarity of the disease and the intention to conduct the analysis within a single center, thereby ensuring greater homogeneity of the case series, made this methodological approach necessary.

The study was conducted in accordance with International Conference on Harmonization Guidelines on Good Clinical Practice and the principles of the Declaration of Helsinki and all available patients signed a written informed consent for the approval of retrospective data collection.

### Procedures and drug administration

All episode were treated starting PEX with solvent-detergent viral-inactivated plasma as soon as the diagnosis of iTTP was confirmed. The volume exchanged was 1.5 times the predicted plasma volume for the first procedure and 1.0 the predicted plasma volume for the following procedures. Daily PEX courses were continued until a clinical response was achieved, defined as resolution of symptoms and a platelet count greater than 150 × 10^9^/L for at least two consecutive days. All received corticosteroid treatment with methylprednisolone/prednisone at the dose of 1 mg/kg/day from diagnosis for 3–4 weeks until remission, followed by gradual tapering until definitive interruption. Rituximab was started in case of suboptimal, delayed or lack of response with a weekly schedule of four infusions at the dose of 375 mg/m^2^. As recommended, caplacizumab 10 mg was administered intravenously before the first PEX course and then subcutaneously every day until 30 days after PEX suspension. Caplacizumab has been used in our center as part of routine clinical practice since late 2020, following approval by the Italian medicines regulatory authority.

## Statistics

Descriptive statistical analyses were used to summarize the data. Quantitative variables are presented using median and interquartile range (IQR), and categorical variables are presented using counts and percentages. The study population was evaluated based on each iTTP acute episode using two categories based on caplacizumab use (CAPLA versus SOC) and disease stage (newly-diagnosed versus relapse). Between-group comparisons were undertaken using an independent Mann-Whitney test for continuous data and Fisher’s exact test for binary variables, with p < 0.05 resulting as statistically significant. The iKaplan-Meier estimator was used to calculate median follow-up time and relapse-free survival (RFS) based on caplacizumab status. All analyses were carried out using MedCalc® Statistical Software version 20.218 (MedCalc Software Ltd., Ostend, Belgium).

## Results

### Baseline iTTP characteristics and clinical presentation

Eighty-one patients were included and the baseline characteristics are reported in Table 1. The median age at first episode was 39.9 years (range 13–75), and 63% were female. The most frequent clinical conditions associated were a personal history of autoimmune disorders, vaccine administration within 2 months, and pregnancy in 25.6%, 8.5% and 2.4%, respectively. Patients presented at diagnosis with a median platelet count of 14 × 10^9^/L, Plasmic score of 6, ADAMTS13 level <1% (range 0–10), and a median ADAMTS13-inhibitor of 49 U/mL (range 2–111) (available in 40% of patients). The signs and symptoms present at disease onset were neurological (59.7%), cutaneous (32.9%), systemic (17.1%), renal (13.4%), and cardiovascular (4.8%). Cutaneous symptoms included hematomas, ecchymosis and petechiae; while systemic symptoms were fever and general malaise.

**TABLE 1:** Baseline characteristics of patients (no. 82).

Baseline characteristics	N = 82
Idiopathic TTP, n (%)	81 (98.7%)
Median age (range)	39,9 (13–75)
Females, n (%)	50 (62.2%)
Males, n (%)	31 (37.8%)
Median follow-up	78,4 months
TTP relapse, n (%)	45 (54.9%)
TTP exacerbation, n (%)	5 (6%)
Comorbidities	
Autoimmune disorders n (%)	21 (25.6%)
Pregnancy n (%)	1 (1.2%)
Vaccine administration <2 months n (%)	7 (8.5%)
Presentation symptoms	
Neurological, n (%)	49 (59.7%)
Renal, n (%)	11 (13.4%)
Fever, n (%)	14 (17.1%)
Cutaneous bleeding, n (%)	27 (32.9%)
Cardiovascular, n (%)	4 (4.8%)
Median PLT count (range)	14 × 10^9/mmc (3–126 × 10^9/mmc)
Median Hb level (range)	8 g/dL (4–14 g/dL)
Median Plasmic Score (range)	6 (3–7)
Median ADAMTS13 activity level	0% (0–10)
Median Inhibitor level (range)	63.2 U/mL (2–111)

TTP, thrombotic thrombocytopenic purpura; PLT, platelets; Hb, hemoglobin.

### Treatment characteristics and outcome based on caplacizumab status

All patients were treated with PEX and corticosteroids at diagnosis, while caplacizumab was administered in 18 patients alone due to previous lack of availability (Table 2). After a median follow-up of 78.4 months, 47 patients (58%) experienced at least one relapse (range 1–7) of iTTP.

**TABLE 2:** Treatment received and days of hospitalization at diagnosis.

First line treatment	​
PEX, n (%)	82 (100%)
Median n. PEX (range)	10 (4–41)
Pre-emptive Rituximab, n (%)	21 (25.6%)
Rituximab, n (%)	7 (8.5%)
Vincristine, n (%)	1 (1.2%)
Corticosteroids, n (%)	82 (100%)
Caplacizumab, n (%)	18 (21.9%)
Median hosptalization days (range)	13 (6–65)

Legend: PEX, plasma exchange procedure.

Every acute episode of iTTP (considering both the onset and the relapse of the disease) was considered apart, obtaining a total of 165 acute episodes. These were analyzed according to caplacizumab administration. Thirty-nine cases (25.5%) were treated with caplacizumab (the CAPLA cohort), while the other 126 cases (74.5%) received the PEX and corticosteroids alone (the SOC cohort). There were no clinically significant bleeding complications associated to the use of caplacizumab, confirming a good safety profile.

**FIGURE 1:**
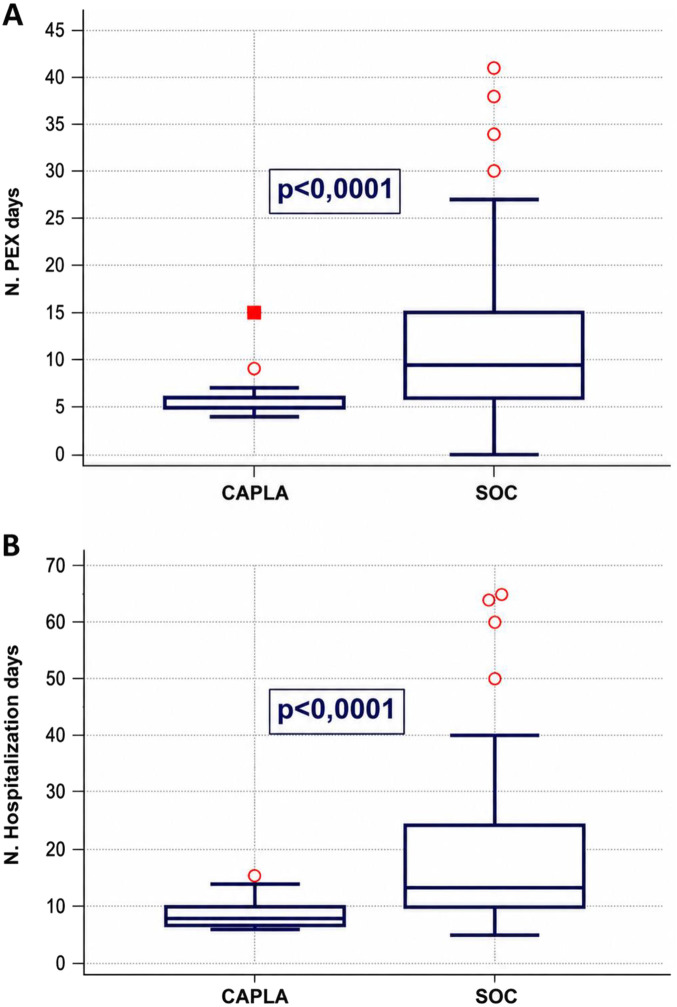
**(A,B)** Median number of PEX days required until platelets normalization (platelets >150 × 10^9/L for two consecutive days) **(A)** and median days of hospitalization **(B)** after acute iTTP episode in patients treated with (CAPLA) and without caplacizumab (SOC) (respectively 6 vs. 9.5 PEX procedures **(A)**, p < 0.0001, and 8 vs. 13.5 days, p < 0.0001 **(B)**) using independent Mann-Whitney test Legend: PEX–plasma exchange; CAPLA (caplacizumab); SOC (standard of care–PEX and corticosteroids).

**FIGURE 2:**
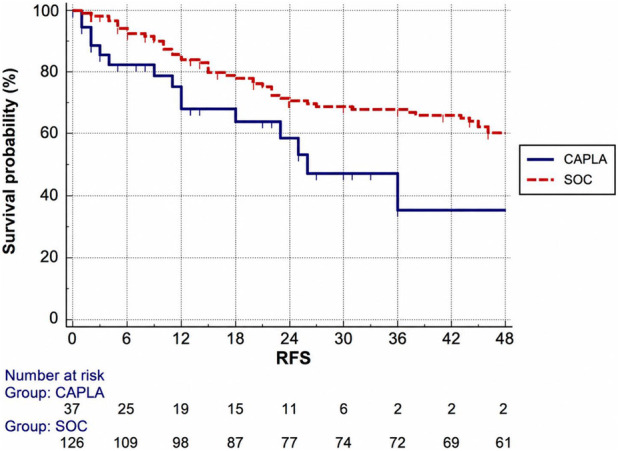
Relapse-free survival of iTTP episodes based on treatment with and without caplacizumab using Kaplan-Meier test (p = 0.1) Legend: RFS (relapse-free survival); CAPLA (caplacizumab); SOC (standard of care–PEX and corticosteroids).

Baseline parameters between the two cohorts were comparable (Table 3). The median number of PEX required before confirmed platelet count normalization for two consecutive days was six for the caplacizumab cohort and was significantly shorter than SOC cohort (median 9.5 PEX sessions, p < 0.0001, Figure 1A), as well as days of hospitalization, that were 8 and 13.5, respectively (p < 0.0001, Figure 1B, using Mann-Whitney test. Relapse-free survival using Kaplan-Meier was longer for SOC cohort (median 80 months vs. 26 months, Figure 2), although it remained non significant (p = 0.11) and the patients treated with CAPLA had a much shorter overall follow-up.

**TABLE 3:** Characteristics of 165 iTTP acute episodes based on caplacizumab status (39 episodes treated with caplacizumab and 126 episodes treated with SOC and statistical analysis using Mann-Whitney and Fisher’s exact test.

Baseline characteristics	CAPLA, value	SOC, value	p
​	N = 39	N = 126	​
Median Age (range)	45.1 (17–75)	43.4 (13–69)	0.37
Females, n (%)	19 (48.7%)	86 (68.2%)	NA
Males, n (%)	20 (51.3%)	40 (31.8%)	
iTTP relapse status n (%)	15 (38.4%)	69 (54.8%)	*0.09*
iTTP early relapse exacerbation, n (%)	5 (12.8%)	6 (4.8%)	0.13
Median PLT count	11 × 10^9^/mmc	18 × 10^9^/mmc	NA
Median Plasmic Score (range)	6 (6–7)	6 (6–7)	NA
Median ADAMTS13 activity level (range)	0% (0–10) [34 cases]	0% (0–10) [58 cases]	NA
Median Inhibitor level, U/mL (range)	66.9 (2–100) [32 cases]	43 (1.86–111) [38 cases]	0.18
Treatment			
PEX, n (%)	39 (100%)	126 (100%)	NA
Median n. PEX (range)	7 (2–41) [34 cases]	10 (2–41) [96 cases]	**<0.0001**
Rituximab, n (%)	6 (15.4%)	11 (8.7%)	0.11
Corticosteroids, n (%)	39 (100%)	126 (100%)	NA
Median hospitalization days (range)	8 (5–65) [30 cases]	20 (6–65) [62 cases]	**<0.0001**
Days to biochemical response (range)	54 (10–228) [18 cases]	39 (28–92) [40 cases]	0.86
Days to platelets count normalization (range)	6 (1–40)	9 (2–40)	NA

iTTP, immune thrombotic thrombocytopenic purpura; CAPLA, caplacizumab; SOC, standard of care treatment–PEX and corticosteroids; PLT, platelet; PEX, plasma exchange procedure; NA, not available. Statistically significant results are highlighted in bold.

Outcomes were defined in accordance with those proposed in 2017 and subsequently revised in 2021 b y the International Working Group for TTP (Cuker et al., 2021).

Exacerbation, defined ad platelet count decrese to <150 × 10^9^/L with or without clinical evidence after a clinical response and within 30 days of stopping PEX (or caplacizumab therapy, if received), occurred in 11 patients: the exacerbation rates were 13% and 5% in patients treated with or without caplacizumab, respectively. Notably, exacerbation was associated with a higher inhibitor level (>66 U/mL) considering all patients (p = 0.03) using ROC analysis and Fisher’s exact test, and although the median inhibitor levels were higher in the CAPLA cohort (67 U/mL vs. 43 U/mL), this data was not statistically significant for patients treated with caplacizumab (p = 0.18). The mean level of ADAMTS13 activity at exacerbation in the CAPLA cohort was <10%, although several patients were on course of caplacizumab at time of the exacerbation.

Biochemical remission, with at least partial ADAMTS13 recovery (>20%), was demonstrated in 36% of acute clinical iTTP episodes with clinical response, respectively 59 out of 165 episodes following a median time of 38 days (range 7–400 days). The probability of achiving biochemical remission was similar in CAPLA cohort compared to SOC, respectively 49% vs. 48%, although ADAMTS13 testing and regular monitoring were available especially in the CAPLA group.

Rituximab was used due to lack of response after the first line treatment in 13% of patients in the CAPLA cohort, compared to 9.5% of SOC patients. The drug was also used in case of exacerbation, respectively in 10% and 1% for patients previously treated with or without caplacizumab. There was also a difference between relapse status based on treatment, although it was not statistically significant (0.09).

Seven patients died (9%) during the follow-up at median age of 50 years (range 34–70), with only two of them (2.5%) following disease relapse and SOC treatment (both without caplacizumab). The others died due to causes not directly related to iTTP.

### Treatment characteristics and outcome based on relapse status

The patients were also classified based on relapse status following SOC treatment with or without caplacizumab and the characteristics are described in Table 4. There were 81 iTTP episodes that remained in clinical remission at last follow-up, while in 84 cases the patients relapsed subsequently. Comparing the two cohorts, the patients that suffered from subsequent relapse had lower median inhibitor level (34 U/mL vs. 63 U/mL, p = 0.045), but longer hospitalization days (12 vs. 10, days p = 0.03) and less frequent caplacizumab use (18% vs. 30%). The prior treatment with caplacizumab was also close to significance in terms of relapse risk (15 vs. 24, p = 0.1). This data is in contrast with a higher incidence of exacerbation for caplacizumab-treated patients.

**TABLE 4:** Characteristics of 165 iTTP acute episodes based on relapse status divided between 84 cases with subsequent relapse and 81 episodes that did not relapse during follow-up and statistical analysis using Mann-Whitney and Fisher’s exact test.

Baseline characteristics	Relapse, value	No relapse, value	p
​	N = 84	N = 81	​
Median Age (range)	43.5 (17–75)	44.4 (13–75)	0.653
Females, n (%)	54 (64.3%)	51 (63%)	NA
Males, n (%)	30 (35.7%)	30 (37%)	
TTP early relapse, n (%)	8 (9.5%)	3 (3.7%)	0.21
Median PLT count	20 × 10^9^/mmc	19.5 × 10^9^/mmc	NA
Median Plasmic Score (range)	6 (6–7)	6 (6–7)	NA
Median ADAMTS13 activity level (range)	0% (0–10) [21 cases]	0% (0–10) [53 cases]	NA
Median Inhibitor level, U/mL (range)	34 (1–93) [22 cases]	63 (2–111) [48 cases]	**0.045**
Treatment			
PEX, n (%)	84 (100%)	81 (100%)	NA
Median n. PEX (range)	8 (2–41) [67 pts]	7 (2–30) [65 cases]	0.298
Caplacizumab, n (%)	15 (17.9%)	24 (29.6%)	0.1
Rituximab, n (%)	6 (7.1%)	11 (13.6%)	0.319
Corticosteroids, n (%)	84 (100%)	81 (100%)	NA
Median hospitalization days (range)	12 (6–65) [24 cases]	10 (5–60) [51 cases]	**0.03**
Days to biochemical response (range)	31 (7–228) [27 cases24]	41 (33–400) [28 cases]	0.176

iTTP, immune thrombotic thrombocytopenic purpura; PLT, platelet; PEX, plasma exchange procedure; NA, not available. Statistically significant results are highlighted in bold.

## Discussion

Immune thrombotic thrombocytopenic purpura is a medical emergency that requires prompt diagnosis and treatment to reduce mortality and avoid critical and potentially lethal consequences (Lewis et al., 2009). For decades the standard of care was the combination of daily PEX procedures and immunosuppressive therapy with corticosteroids (Kremer Hovinga et al., 2017). This treatment protocol was recently modified after the introduction of caplacizumab, leading a major improvement in patient management and survival. Caplacizumab was approved by European Medicine Agency and US Food and Drug Administration between 2018 and 2019, following the publication of phase two TITAN (2016) and phase three HERCULES (2019) randomized clinical trial (RCT) results (Peyvandi et al., 2016; Scully et al., 2019). These studies demonstrated the efficacy of caplacizumab in terms of faster platelet count recovery and fewer exacerbations compared to the standard of care. However, the RCTs did not show a reduction in mortality, although this was not their primary end-point. Several real-world analyses have been published since its approval, confirming the safety and efficacy of caplacizumab in combination with PEX and corticosteroids alone (Table 5). In a recent meta-analysis of both RCTs and observational studies, a nonsignificant reduction in the relative risk of death was shown with caplacizumab compared to SOC (Djulbegovic et al., 2023; Goshua et al., 2021). Other recent systematic reviews and meta-analyses have confirmed the promising results showed in two registrative trials, in terms of rapid platelet response and disease remission along with lower rates of exacerbations and lower mortality in patients with iTTP, with an overall favorable safety profile (Peng et al., 2024; Yassin et al., 2025).

**TABLE 5:** Real-world study about caplacizumab use in iTTP patients.

Study	Völker et al. (2020b)	Coppo et al. (2021)		Dutt et al. (2021)		Izquierdo et al. (2022)	
Country	Germany	French		United Kingdom		Spain	
Treatment group	CAPLA	CAPLA	SOC	CAPLA + Rituximab	SOC	CAPLA	SOC
Patients	60	90	180	85	39	77	78
Characteristic							
Mean age (range) – years	45.7 (22–83)	45 (34–57)	43 (30–57)	46 (3–82)	45 (15–93)	47.1 ( ± 14)	46.5 ( ± 14)
Female sex - no. (%)	42 (70)	63 (70)	127 (70)	56 (66)	31 (80)	58 (75)	61 (78)
Median platelet count (range) - x10^9	13 (3–85)	12 (10–20)	12 (8–23)	13 (9–21)	10 (6–20)	12 (8–20)	12 (8–17.5)
ADAMTS13 activity	<10%, 60 (100)	<10%	<10%	<10%, 84 (99)	<10%, 39 (100)	0 (0–0.5)	0 (0–0.5)
ADAMTS13 inhibitor (BU)	​	​	​	​	​	3.3 (7–20.9)	NA
ADAMTS13 antibodies (U/mL)	75.5 (0–131.6)	78 (39–91)	80 (36–100)	​	​	​	​
Symptoms/signs							
Neurologic	​	55 (61)	111 (62)	56 (66)	29 (74)	43 (55.8)	47 (60.3)
Cutaneous	​	​	​	​	​	39 (50.6)	18 (23.1)
Renal	​	​	​	10 (26)	35 (41)	19 (24.7)	19 (25.0)
Cardiac	27 (84.4)	51 (56)	86 (47)	25 (64)	67 (79)	22 (50.0)	NA
Therapy							
Glucocorticoids	59 (98.3)	88 (98%)	166 (92%)	84 (99)	​	​	​
Rituximab	47 (78.3)	90 (100%)	123 (68%)	84 (99)	34 (87)	65 (84)	53 (68)
Outcome							
Death rate	​	1 (1.1)	12 (6.7)	5 (6)	0 (0)	2 (4.5)	6 (7.7)
Median platelet recovery - days	3 (1–13)	5 (4–6)	12 (6–17)	4 (3–8)	6 (4–10)	​	​
Median PEX number	4 (0–22)	5 (4–7)	10 (6–16)	7 (5–14)	9 (8–16)	8.5 (6–12.5)	14 (7–21.5)
Median days of hospetalization	18 (5–79)	13 (9–19)	22 (15–30)	12 (8–24)	14 (9–17)	12 (9–15)	19 (12–27)
Exacerbation	2 (3.3)	3 (3.4)	70 (44)	2 (2)	​	2 (4.5)	16 (20.5)

Study	Völker et al. (2023)		Agosti et al. (2023)		Angelucci et al. (2024)	Gavriilaki et al. (2023)	
Country	Greece		Germany		Italy	Italy	
Treatment group	CAPLA	SOC	CAPLA	SOC	CAPLA	CAPLA	SOC
Patients	23	47	113	119	26	42	168
Characteristic							
Mean age (range) - years	47 (24–74)	45 (19–85)	46	46	53 (47.3–60.4)	48.5 (40.2–53.8)	48.5 (36–57)
Female sex - no. (%)	15 (65)	33 (71)	77 (70)	63 (66)	20 (76.9)	31 (69)	115 (68.5)
Median platelet count (range) - x10^9	23 (3–115)	21 (5–100)	13 (8–22)	16 (11–32)	14 (10–19)	​	​
ADAMTS13 activity	​	​	110 (97)	93 (91)	​	​	​
ADAMTS13 inhibitor (BU)							
ADAMTS13 antibodies (U/mL)							
Symptoms/signs							
Neurologic	50%	53%	​	​	15 (57.7)	​	​
Cutaneous	​	​	​	​	12 (46.2)	​	​
Renal	​	​	​	​	4 (15.4)	​	​
Cardiac	31%	43%	52 (79)	44 (80)	5 (19.2)	​	​
Therapy							
Glucocorticoids	100%	100%	111 (99)	117 (98)	​	​	​
Rituximab							
Outcome							
Death rate							
Median platelet recovery - days	​	​	12 (4–24)	12 (6–20)	4 (3–4)	0 (0)	17 (10.1)
Median PEX number	12 (8–23)	14 (6–32)	7 (4–12)	8 (5–14)	7 (6–14)	​	​
Median days of hospetalization	​	​	17 (12–25)	18 (11–25)	18 (9–29)	13 (9–17)	15 (7–30)
Exacerbation	0 (0)	​	46 (41)	49 (43)	1 (4.3)	​	​

PEX, plasma exchange procedure; CAPLA, caplacizumab; SOC, standard of care.

In the present study, we aimed to evaluate the impact of caplacizumab in the treatment of iTTP, by comparing single episodes treated with caplacizumab versus SOC before its approval or in case of contraindication, both at disease diagnosis and after clinical relapse. Notably, the study cohort had similar characteristics to those described in other iTTP studies. As expected, most patients were female, in their fourth to fifth decade of life, mostly presenting with neurologic symptoms or cutaneous bleeding. We confirmed that caplacizumab is effective in terms of speed of clinical response, based on the reduction in the number of daily PEX required, due to a more rapid time to platelets normalization. Similarly, caplacizumab is associated with a shorter period of in-patient hospitalization. Both findings are consistent with RCTs and other real-world analyses, confirming the efficacy of caplacizumab. Since the drug’s approval, only one patient in our cohort did not receive caplacizumab due to a clinical contraindication, as she was pregnant at the time of diagnosis. The patient did not received caplacizumab due to the lack of safety data during pregnancy. Another patient was diagnosed 5 days after delivery, but she was not treated with caplacizumab due to unavailability of the drug at the time of the episode.

In our retrospective study patients treated with caplacizumab had rate of biochemical remission comparable to patients treated without caplacizumab with ADAMTS13 activity greater than 20%, respectively 49% vs. 48%. However, the lack of availability of ADAMTS13 evaluation in the initial SOC patients and the possibility of ADAMTS13 monitoring and the use of pre-emptive rituximab treatment in the last 10–15 years had a major impact in this retrospective setting, thus biochemical remission rates cannot be estimated in a precise manner between the two study groups.

In contrast to previously reported studies, we observed an increased risk of exacerbation in episodes treated with caplacizumab, although not statistically significant. Exacerbation is considered as the recurrence of the disease within 30 days from PEX suspension. The condition was associated with an ADAMTS13 inhibitor titer higher than 66 U/mL for the entire study cohort, and although the inhibitor levels were higher for the CAPLA group, this data was not statistically significant. Unfortunately, data on the inhibitor are missing for a substantial proportion of patients due to the unavailability of the test at the time of diagnosis or during exacerbation/relapse. This represents a limitation to the statistical significance of the study. A systematic analysis of inhibitor levels could, in fact, clarify whether there is an association between high inhibitor levels and the risk of exacerbation/relapse, as well as whether there is a correlation between inhibitor levels and the use of caplacizumab. Increased risk of exacerbation in CAPLA cohort may be explained because caplacizumab does not actually correct the underlying ADAMTS13 deficiency, and in case of high inhibitor titer additional PEX sessions might prove beneficial in reducing early relapse risk. It is interesting to note that even in cases of exacerbation among patients treated with caplacizumab, the mean ADAMTS13 activity was below 10%. This finding reflects the fact that, to date, the indication for discontinuing caplacizumab therapy was based solely on a time-based criterion in our cohort (30 days after the last PEX) due to local indications and regulatory and budget issues and did not take ADAMTS13 activity levels into account, as recommended (ADAMTS13 activity >20%).

The optimal strategy regarding the use of caplacizumab in patients with iTTP remains under investigation. Alongside the well-established time-based strategy, where caplacizumab is administered until 30 days after PEX cessation, a goal-directed approach is also being evaluated, in which caplacizumab is continued until recovery of ADAMTS13 activity. Observational data from German and Austrian cohorts have demonstrated that serial monitoring of ADAMTS13 activity can guide the optimal duration of caplacizumab therapy and PEX, potentially minimizing both overtreatment and undertreatment while maintaining favorable clinical outcomes. In this study, an ADAMTS13 activity threshold (e.g., >10%) was associated with safe discontinuation of therapy, allowing for a tailored, patient-specific strategy (Völker LA. et al., 2020). Similar findings have been reported in other studies, reinforcing the concept that ADAMTS13-guided management can complement standard clinical and laboratory criteria in TTP (Yates et al., 2024; Palandri et al., 2021; Robert et al., 2025). Notably, the MAYARI trial (NCT05468320), a multicenter Phase III study, is currently evaluating caplacizumab in combination with immunosuppressive therapy without first-line plasma exchange, with ADAMTS13 activity recovery (≥50%) used as a primary criterion for treatment continuation and endpoint assessment. It is therefore plausible to hypothesize that exacerbation after caplacizumab treatment could have been prevented, at least in some patients, if caplacizumab was continued until at least partial recovery of ADAMTS13 activity or even inhibitor neutralization. It would therefore be interesting to investigate in a prospective clinical study whether continuing caplacizumab treatment until ADAMTS13 activity normalizes or the inhibitor disappears could be associated with improved outcomes, particularly a reduced risk of exacerbation or relapse.

Although it most commonly remains unexplained, the potential cause of both disease onset and relapse, given its autoimmune mechanism, may have a major influence on inhibitor level and response rate. One of the potential triggers remains mRNA COVID-19 vaccination, and it has been described frequently in the last years, including our single-center experience (Giuffrida et al., 2022).

Rituximab, a humanized anti-CD20 monoclonal antibody, was introduced in iTTP treatment in patients with exacerbations or refractoriness to conventional therapy, in order to suppress the B-lymphocytes responsible for anti-ADAMTS13 autoantibody production (Scully et al., 2007). Furthermore, two prospective studies analyzed the use of rituximab as front-line therapy, and showed fewer and delayed relapses (Scully et al., 2011; Froissart et al., 2012). The use of up-front rituximab in combination with PEX and corticosteroids remains debated. The ISTH guidelines for iTTP recommend the use of rituximab in addition to corticosteroids and PEX for patients with their first acute event or relapse. Even though the data regarding rituximab use are from non-randomized trials, it appears that the use of rituximab is associated with a reduced risk of relapses. The panel of ISTH also suggests the pre-emptive rituximab use in patients in clinical response but with persistently low plasma ADAMTS13 activity and high inhibitor titers, i.e., the so-called biochemical relapse. The rates of rituximab use following first-line therapy in non-responsive patients or with early exacerbations were higher in the CAPLA group, possibly due to prolonged persistence of the ADAMTS13 inhibitor owing to the reduced number of PEX procedures. Additionally, the analysis of this data should take into consideration that rituximab has been available for iTTP since the late 2000s, whereas we evaluated patients diagnosed since 1990, so the difference in the use of rituximab firstly reflects on different availability.

In RCTs patients who received caplacizumab presented a clinically and statistically increased relapse rate (defined as disease recurrence after 30 or more days since last PEX procedure) at 12 months. This enforces the possibility that caplacizumab use is not enough in case of extremely active disease in terms of inhibitor level. The relapse-free survival (RFS) in our study was longer for SOC group as expected, given that the first patient was diagnosed in 1990, while the longest remission for CAPLA patient was 24 months. These data could be affected by bias given the retrospective data collection still remain unconfirmed in a significant manner using statistical analysis (p = 0.1). However, even though the probability of suffering from subsequent relapse was associated with a higher inhibitor titer in our study, as expected the relapse risk was lower in the case of caplacizumab use, although it was not statistically significant (p = 0.1). These data suggest that caplacizumab use could possibly confer potential long-term protection when the early relapse window is overcome. However, the data available in the literature thus far are not conclusive for a possible effect of caplacizumab in this regard. In addition, possible factors that may interfere with the interpretation of these data must be considered, such as the use of rituximab. Indeed, the action of rituximab may reduce the risk of relapse, and its use has increased in recent years along with the introduction of caplacizumab into clinical practice.

Even though a retrospective study has significant limitations, our data confirm the effectiveness of caplacizumab in terms of faster platelet recovery, leading to reduced risk in terms of morbidity and complications. Furthermore, caplacizumab diminishes healthcare disease burden, both in terms of days of hospitalizations and number of PEX sessions. The high cost of the drug could be partially recovered by the reduced hospitalization duration, reduced number of PEX required and the time of work assence of patients themselves.

## Conclusion

Here, we report a comparative analysis of iTTP episodes treated with and without caplacizumab at our center. We suggest that caplacizumab significantly reduces the treatment burden of iTTP episodes in terms of the number of PEX procedures and days of hospitalization, confirming faster clinical response and shorter access in the in-patient setting in real-life. This innovative treatment, on the other hand, might potentially lead to higher rates of exacerbations due to premature PEX suspension and more frequent use of rituximab, although the rate following the first 30 days seems to be less frequent.

## Data Availability

The raw data supporting the conclusions of this article will be made available by the authors, without undue reservation.
